# Antiretroviral drug expenditure, pricing and judicial demand: an analysis of federal procurement data in Brazil from 2004–2011

**DOI:** 10.1186/1471-2458-14-367

**Published:** 2014-04-16

**Authors:** Jing Luo, Maria A Oliveira, Mariana BC Ramos, Aurélio Maia, Claudia GS Osorio-de-Castro

**Affiliations:** 1 Department of Internal Medicine, Yale-New Haven Hospital, New Haven, USA; 2 Sergio Arouca National School of Public Health, Oswaldo Cruz Foundation, Rio de Janeiro, Brazil; 3 Ministry of Health, Brasilia, Brazil

**Keywords:** Antiretroviral treatment, Drug procurement, HIV/AIDS, Brazil, Judicial demand, Access to medicines

## Abstract

**Background:**

Previous studies have described expenditures for antiretroviral (ARV) medicines in Brazil through 2005. While prior studies examined overall expenditures, they have not have analyzed drug procurement data in order to describe the role of court litigation on access and pricing.

**Methods:**

ARV drug procurement from private sector sources for the years 2004–2011 was obtained through the general procurement database of the Brazilian Federal Government (SIASG). Procurement was measured in Defined Daily Doses (DDD) per 1000 persons-under-treatment per day. Expenditures and price per DDD were calculated and expressed in U.S. Dollars. Justifications for ARV purchases were examined in order to determine the relationship between health litigation and incorporation into Brazil’s national treatment guidelines.

**Results:**

Drug procurement of ARVs from private sources underwent marked expansion in 2005, peaked in 2009, and stabilized to 2008 levels by 2011. Expenditures followed procurement curves. Medications which were procured for the first time after 2007 cost more than medicines which were introduced before 2007. Judicial actions initially resulted in purchases of newer medications for a select number of patients in Brazil but ultimately expanded availability to a larger population through incorporation into the national treatment guidelines.

**Conclusions:**

Drug procurement and expenditures for ARVs in Brazil varied between 2004–2011. The procurement of some drugs from the private sector ceased after public manufacturers started producing them locally. Judicial demand has resulted in the incorporation of newer drugs into the national treatment guidelines. In order for the AIDS treatment program to remain sustainable, efforts should be pursued to reduce prices through generic drugs, price negotiation and other public health flexibilities such as compulsory licensing.

## Background

Brazil is a middle-income country that has officially provided universal access to anti-retroviral treatment since 1996 [1]. In what has become known as the “Brazilian Model,” the national AIDS program simultaneously balanced the need for expanded access with the needs of program sustainability [2-4]. Although patents for pharmaceutical products have been granted since 1997, authorities have been able to utilize public health flexibilities in order to decrease costs associated with treatment [5,6]. For example, between 2000 and 2004, overall expenditures for antiretroviral medications (ARVs) decreased, despite an increase in the number of people receiving ARVs. This was mostly due to generic competition, negotiated price reductions with originator companies, and domestic production through Brazil’s public drug manufacturers [7]. However, by 2005, changes to first and second line treatment guidelines and the introduction of newer, patented medicines led to an increase in expenditures [8,9] and to an upsurge in judicial demand for originator medicines which were previously unavailable through Brazil’s national treatment guidelines. Between the years 2007 and 2009, treatment costs decreased from 2005 levels, and remained around $1700 per patient per year [10]. In 2011, there were approximately 216,000 people on treatment, representing an estimated ART coverage of 72% [11].

Using the rhetoric of human rights and anti-discrimination, civil society groups in Brazil have been instrumental in advancing the access agenda [12]. A compulsory license was issued for efavirenz in 2007, which reduced treatment expenditures by approximately $103.6 million [13]. Gilead’s initial patent for tenofovir was rejected after civil society groups filed a successful pre-grant opposition [14]. Patient advocacy groups have also been successful in using Brazilian courts to win access to new, previously unavailable medications [15].

Due to administrative changes, official data on expenditures for AIDS treatment has not been easily accessible. Since the 2011 passage of an access to information law (No. 12.527), alternate sources of government procurement data have become available. The objective of our study was to describe the evolution of private sector ARV procurement and expenditures from 2004–2011 using data from the federal government. We also describe judicial actions for newer ARVs, examining their results on increased availability.

## Methods

ARV drug procurement from the private sector for the years 2004–2011 was obtained from the *Sistema Integrado de Administração de Serviços Gerais (SIASG)*, the general procurement database of the Brazilian federal government. The SIASG data is publically available; however availability is subject to data extraction by the Department of Health Economics, Investment, and Development at the Ministry of Health. This database includes only purchases from the private sources (both national and international suppliers), and does not include medicines procured from local public manufacturers such as Farmanguinhos. Filters were used to select data specific to the Ministry of Health and the Logistics Department (who is solely responsible for purchases for all ARVs). A comprehensive list of all purchases of ARVs from the private sector was thus obtained for each year. Information was obtained for the following variables: name of drug, dosage form and concentration, quantity, unit price, date of purchase, justification for purchase and method of tender.

ARVs were then classified by means of the WHO Anatomical Therapeutic Chemical Classification System (ATC) and their Defined Daily Doses (DDD) obtained [16,17]. In order to make this unit of measurement more adequate for the description of ARV procurement, the number of DDDs was calculated for each ARV and the results expressed as the number of DDDs/1000 persons-under-treatment/day. We chose to express volume procured in this manner because it reflects the extent to which procurement from private sector sources satisfies demand for ARVs in Brazil. Data on volumes and expenditures from public sector procurement was not available due to the nature of the SIASG database. The number of patients on treatment nationally was obtained from the MonitorAIDS website for the years 2004–2010 [18]. The number of people on treatment in 2011 was obtained from the national program for STD/AIDS (personal communication).

Subgroup analyses also included collapsing the ARV ATC codes to map procurement over time according to antiretroviral treatment class. The justification for each ARV purchase was examined in order to determine the dynamics of purchases in relation to health litigation. We then plotted the number of purchases which resulted from court cases over time and compared these actions with both bulk purchases and the dates of incorporation of each medicine into the national treatment guidelines [19].

We expressed prices of individual drugs using price per DDD (in U.S. Dollars). The price per DDD is a better approximation of treatment prices than price per tablet because the DDD is based on the average adult daily dose. To calculate price per DDD, we summed each ARV in grams and then divided that sum by the listed DDD for the particular drug to obtain the total number of DDDs purchased. We then divided the total amount spent for that drug in a given year by the total number of DDDs to obtain price per DDD.

We show individual drug pricing for select ARVs only from 2007 onwards because many novel ARVs were not procured in Brazil prior to 2007, making it difficult to compare prices of ARVs from 2004 to 2007. Furthermore, 2007 was a landmark year in Brazil’s national treatment program because of the issuance of a compulsory license for efavirenz.

Calculations and graphs were made with the help of Excel (Microsoft Corp. 2010). Expenditures were calculated by multiplying unit price by volume purchased. Costs were expressed in U.S. Dollars using mean annual exchange rates provided by the U.S. Federal Reserve Bank [20].

## Results

**Figure 1:**
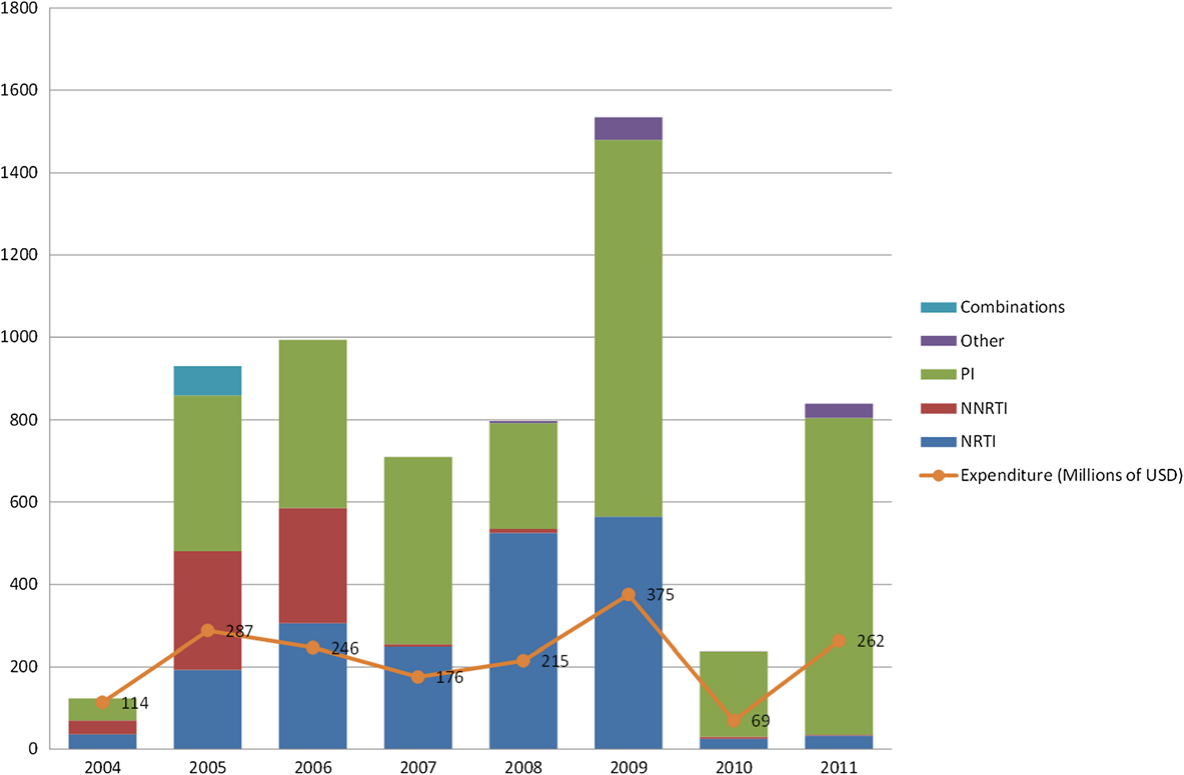
**Drug Procurement and total expenditures for antiretroviral medicines in Brazil from 2004–2011.** Volume procured expressed as DDD/1000 persons-under-treatment/day.

Overall, our database of ARVs included 21 different medications in 40 dosage forms. There were only two fixed dose combination ARV medications: lopinavir/ritonavir and zidovudine/lamivudine. Individual purchases ranged from as few as three units (tipranavir in 2006) to as many as 106,080,000 units (lopinavir 200 mg/ritonavir 50 mg in 2011). The results of our descriptive analysis of the SIASG database are shown in Figure 1. This figure shows drug procurement from the private sector expressed in number of DDDs/1000 persons-under-treatment/day according to WHO ATC therapeutic class. It also shows annual federal expenditures for privately procured ARVs in U.S. dollars from 2004 – 2011.

### Procurement in DDDs per 1000 persons under treatment per day

Overall drug procurement rose dramatically from a low of 124 DDDs/1000 persons-under-treatment/day in 2004 to 929 DDDs/1000 persons-under-treatment/day in 2005. While procurement from the private sector involved all three major classes: nucleoside reverse transcriptase inhibitors (NRTIs), non-nucleoside reverse transcriptase inhibitors (NNRTIs), and protease inhibitors (PIs) from the years 2004 until 2006, by 2007 the procurement of NNRTIs fell drastically and PIs became the predominant class of medications procured from the private sector by the federal government. This trend continued in subsequent years (with the exception of 2008, where a large purchase of tenofovir resulted in higher number of DDDs/1000 persons-under-treatment/day of NRTIs when compared to PIs). In 2008, the government began to procure newer classes of drugs such as integrase inhibitors (raltegravir), entry inhibitors (maraviroc) and fusion inhibitors (enfurvitide). In 2010, there was a significant reduction in overall drug procurement from the private sector (from 1534 DDDs/1000 persons-under-treatment/day to 238 DDDs/1000 persons-under-treatment/day), which was likely due to the fact that some intensively purchased or expensive medications such as lopinavir/ritonavir and darunavir were possibly procured in excess quantities during the previous year in order to cover the needs of the national treatment program for a two year span (data not shown). Additionally, the NRTI tenofovir (which represented the vast majority of NRTIs utilized in prior years) was not purchased from the private sector in either 2010 or 2011 due to domestic production in Brazil’s national public laboratories.

In 2004, the predominant PI procured was saquinavir. In 2005 and 2006, the most frequently procured PIs were atazanavir (362 DDDs/1000 persons-under-treatment/day) and nelfinavir (14 DDDs/1000 persons-under-treatment/day). In 2007, lopinavir/ritonavir became predominant PI (306 DDDs/1000 persons-under-treatment/day). This was also the case for the years 2009 and 2011. On alternating years (2008 and 2010), atazanavir was the most frequently procured PI.

### Expenditures

The curve of federal expenditures follows that of drug procurement (Figure 1). Overall federal expenditures for private sector ARV procurement more than doubled from $114 million dollars in 2004 to $287 million dollars in 2005. Expenditures then came down slowly over the next three years (with a low of $176 million dollars in 2007). In 2009, federal expenditures for ARVs again doubled to reach a high of $375 million dollars before falling to $69 million dollars in 2010. In 2011, expenditures returned to 2006 levels ($262 million dollars).

### Pricing

In general, between 2007 to 2011, the price of medications per DDD were higher for drugs which were added to Brazil’s national treatment guidelines more recently when compared to older ARVs (see Table 1). For example, on average, medications which were introduced prior to 2007 (e.g. didanosine, saquinavir) had price per DDD ranging from $1.55 to $7.98. Medications procured more recently such as raltegravir and maraviroc had price per DDD ranges between $18.88 and $33.02.

**Table 1:** Price per DDD for selected ARVs between 2007 and 2011

	**2007**	**2008**	**2009**	**2010**	**2011**	**Average**
Abacavir	4.62	2.94				3.78
Didanosine	1.54		1.73	1.51	1.41	1.55
Nelfinavir	4.38					4.38
Saquinavir	7.20	7.80		8.23	8.68	7.98
Atazanavir	3.51	4.13	4.86	3.92	3.11	3.91
Lopinavir/ritonavir	2.79		2.92		2.31	2.67
Darunavir	19.79	19.92	15.86		16.60	18.04
Etravirine		30.95		22.72	22.96	25.54
Raltegravir		21.31	18.48		16.85	18.88
Maraviroc		25.27	29.90	37.00	39.90	33.02

All values expressed as $U.S. Dollars.

Some medications experienced significant price reductions over time. For example, Brazil paid $80.06 per DDD for lopinavir 133 mg/ritonavir 33.3 mg capsules in 2004. By 2011, the government of Brazil purchased only the 200 mg/50 mg heat-stable formulation of lopinavir/ritonavir at a cost of $2.31 per DDD. While the exact rationale for this thirty-fold reduction in price per DDD cannot be derived from the SIASG database, our hypothesis is that Brazil’s involvement in an international movement demanding access to ARVs and the threat of a compulsory license for lopinavir/ritonavir allowed the Ministry of Health to negotiate significant price reductions for this medication with Abbott Laboratories [5].

### Expanding access to treatment, the relationship between judicial action and incorporation into national treatment guidelines

**Figure 2:**
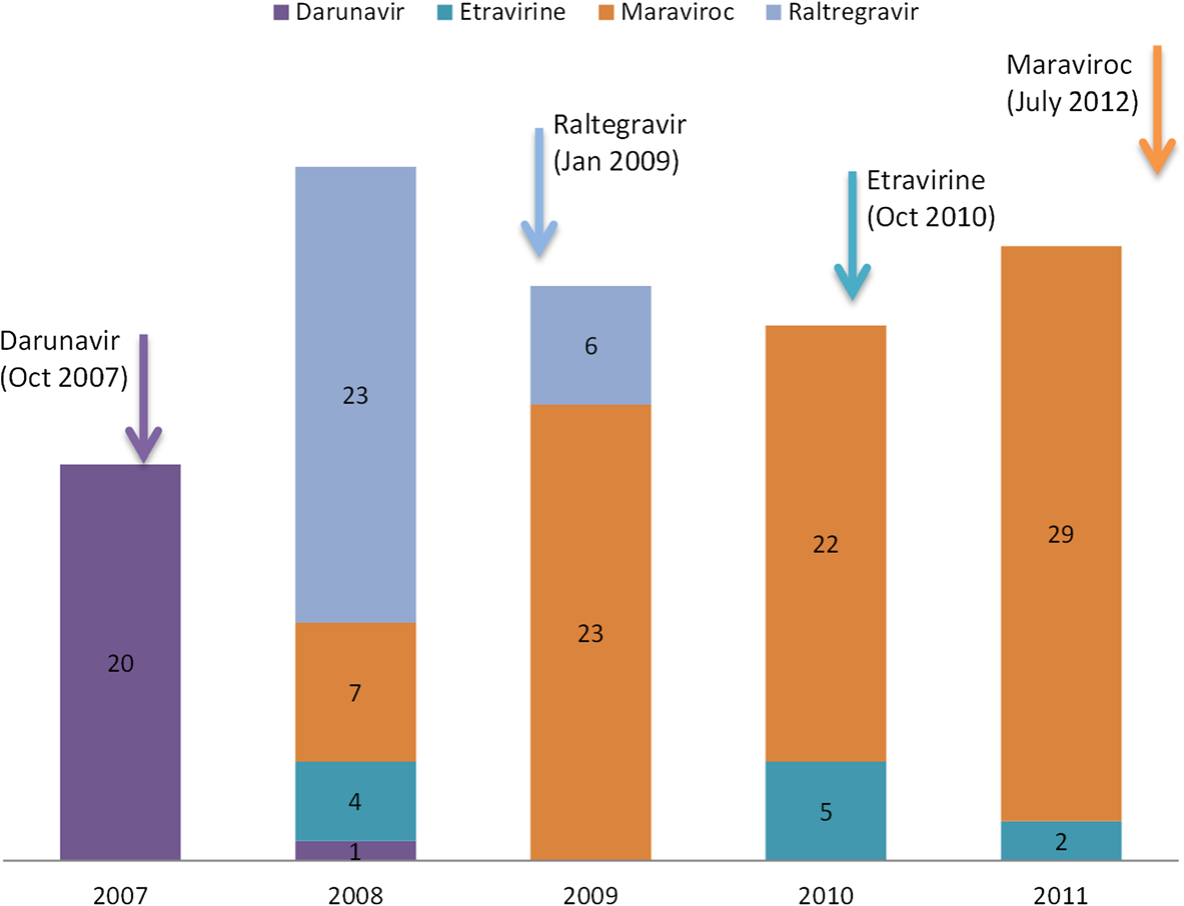
**Number of purchases of selected antiretroviral medications as a result of judicial action, from 2007–2011.** Colored arrows indicate the approximate time when each medication was incorporated into Brazil’s national treatment guideline.

Figure 2 shows the number of purchases per year of four medications (darunavir, etravirine, raltegravir and maraviroc) based on the justification of judicial action. As demonstrated, the overall number of judicial actions increased from 2007 to 2011, with a high in 2008 of 35 judicial actions resulting in ARV purchases.

A higher number of judicial actions for a drug made it more likely for that drug to be subsequently incorporated into the national treatment guidelines. For example, there were 20 judicial actions in 2007 for darunavir between January 7^th^ and December 31^st^ resulting in the purchase of 13,200 units of the drug. Darunavir was added to the treatment guidelines in October 2007. On December 26^th^, shortly after incorporation, the government purchased 2.28 million units of darunavir. After a drug was added to the treatment guidelines, judicial demand falls dramatically. For example, there was only one darunavir purchase due to judicial action in the years following its incorporation. This trend continued for all other ARVs in the database.

There were only 6 judicial actions for raltegravir in 2009. However, in the year prior to its incorporation, there were 23 judicial actions resulting in 9,360 units purchased. On November 18^th^, 2008 there was a purchase of 720,000 units of raltegravir, likely in anticipation of its official incorporation into the consensus guidelines by January 2009.

There were 5 judicial actions in 2010 for etravirine resulting 3,240 units purchased. Etravirine was incorporated into the treatment guidelines on October 2010. On August 12, 2010, the government procured 403,200 units of etravirine.

From 2009 to 2011, there were over 20 judicial actions each year resulting in purchases of maraviroc. In 2011, there were 29 judicial actions for maraviroc resulting in the purchase of 24,600 units of maraviroc. This medication was not included until the most recent supplement to the treatment guidelines (July 2012).

## Discussion

Our study is the first of its kind in Brazil which describes private sector ARV drug procurement from the years 2004–2011. Previous studies used expenditure data provided from the national AIDS program, but were from analysis which did not include line-item data such as price per unit, or justification for purchase. Thus, while prior studies estimated contracted demand, ours reflects actual procurement. Additionally, we were able to examine the effect of litigation on the procurement of newer medications and incorporation into the national treatment guidelines. Our work increases the understanding of Brazil’s evolving national AIDS treatment program, which was founded on the principles of universal access [12,21].

### Implications for pricing and sustainability

Our results show that the pricing of drugs has not remained stable. Medicines which were introduced at very high prices such as and lopinavir/ritonavir achieved significant price reductions over time. In general, ARVs first introduced in Brazil after 2007 cost significantly more per DDD than ARVs introduced prior to 2007.

Our results suggest that the government of Brazil should be prepared for a trend towards the use of newer, more expensive medications. Although these newer medications are reserved for cases of treatment failure or for salvage regimens, the fact that the AIDS population in Brazil is one of the oldest treatment cohorts among developing countries suggest that newer medicines will be needed. Our results indicate a trend towards growth in the procurement of fusion inhibitors, integrase inhibitors and newer PIs which have no generic competition. As such, in order for the program to remain sustainable, efforts should be aggressively pursued to reduce prices through price negotiation, exercising TRIPS flexibilities and local production [22].

### Results of Judicial Action

Our results indicate that the number of judicial actions is related to timing of incorporation into Brazil’s national treatment guidelines. With the exception of etravirine (which was incorporated after published data showed improved outcomes for treatment-experienced patients), the government of Brazil usually timed its purchases of large quantities of newer ARVs following the results of numerous court cases, often greater than 20 a year, in favor of plaintiffs [23]. Shortly after these large purchases by the Ministry of Health, the medication was incorporated into the following years’ treatment guidelines.

These results suggest one of three possibilities: 1) the relationship between number of judicial actions and timing of incorporation into the national treatment guideline is coincidental (unlikely), 2) judicial demand is a reflection of established treatment preferences by prescribers (which prompts review by the expert committees who meet annually to draft treatment guidelines), or 3) judicial action may exert a previously undescribed degree of influence on a process which is presumed to be objective and evidence-based.

Our work supports previous literature which has described the impact of litigation for access to medicines [24-27]. While many have suggested that drug companies may be using patient advocacy groups to expand market share through litigation, our study is the first which directly examines the impact of judicial cases on national drug procurement [28]. Overall, our results indicate that judicial demand has been highly successful in granting access to newer ARVs.

### Strengths and Limitations

One of the strengths of our study was that we were able to monitor trends in expenditures for private sector purchases of ARVs by the federal government. These medications often account for the bulk of expenditures for AIDS treatment because they are patented, and have little or no available competitors in the national or international market. Additionally, this type of drug procurement may be a proxy for measuring external dependency in the medicines market as all recorded drugs were purchased either directly from foreign suppliers, or from domestic private pharmaceutical companies operating under licensing agreements with foreign companies. Another strength is that our data is extracted from only one ministry located in the global South, and does not include figures from international sources such as the Global Fund, PEPFAR, or Clinton Health Access Initiative [29].

Limitations of our study include the fact that we could only describe one measure of drug utilization (procurement), and did not have access to other measures such as number of prescriptions or level of consumption. We could not describe relative use of drug classes in the population directly because we did not have access to treatment data. Additionally, because the SIASG database does not include public domestic production, we could not estimate overall ARV procurement as a proxy of drug utilization for the entire country. However, we did validate our data by comparing our expenditures with what was available from the literature and the national AIDS program.

### Future Studies

Our work opens the possibilities for many future studies. For example, the SIASG database could be examined to compare prices paid with patent status of medications in Brazil [30]. Additionally, if this data could be combined with drug procurement measures from public national manufacturers and drug prescribing and dispensing (a proxy to consumption), a correlation would be shown between increased drug utilization and improved clinical outcomes such as decreased AIDS morbidity/mortality and reduced transmission. Another study could further explore the causes for lopinavir/ritonavir’s thirty-fold reduction in price from 2004 to 2007.

## Conclusions

Drug procurement and expenditures for private sector ARVs in Brazil varied between 2004–2011. ARVs which were procured for the first time after 2007 cost more than medications which were introduced prior to 2007. Judicial demand has resulted in the procurement of large quantities of newer, more expensive medications through incorporation into the national treatment guidelines. Our study suggests that recent judicial actions may have an impact on program sustainability. In order for the AIDS treatment program to remain sustainable, efforts should be pursued to reduce prices through price negotiation and other public health flexibilities.

## Competing interests

The authors declare that they have no competing interests. Although the data was provided by the Ministry of Health of Brazil, authors received no additional financial payments for the analysis here and the Ministry had no role in the analysis or interpretation of the data.

## Authors’ contributions

JL participated in the design of the study, the data analysis and draft of the manuscript. MO and CO participated in the design of the study, the acquisition of data, data analysis and draft of the manuscript. MR and AM participated in acquisition of data and participated in the design of the study. All authors read and approved the final manuscript.

## Pre-publication history

The pre-publication history for this paper can be accessed here:

http://www.biomedcentral.com/1471-2458/14/367/prepub
